# Resistance training concomitant to radiotherapy of spinal bone metastases – survival and prognostic factors of a randomized trial

**DOI:** 10.1186/s13014-016-0675-x

**Published:** 2016-07-27

**Authors:** Harald Rief, Thomas Bruckner, Ingmar Schlampp, Tilman Bostel, Thomas Welzel, Jürgen Debus, Robert Förster

**Affiliations:** 1 Department of Radiation Oncology, University Hospital Heidelberg, Im Neuenheimer Feld 400, 69120 Heidelberg, Germany; 2 Department of Medical Biometry, University Hospital Heidelberg, Im Neuenheimer Feld 305, 69120 Heidelberg, Germany; 3 National Center for Radiation Oncology (NCRO), Heidelberg Institute for Radiation Oncology (HIRO), Im Neuenheimer Feld 400, 69120 Heidelberg, Germany

**Keywords:** Bone metastases, Spine, Resistance training, Survival, Palliative radiotherapy

## Abstract

**Purpose:**

To compare the effects of resistance training versus passive physical therapy on bone survival in the metastatic bone during radiation therapy (RT) as combined treatment in patients with spinal bone metastases. Secondly, to evaluate overall survival and progression-free-survival (PFS) as well as to quantify prognostic factors of bone survival after combined treatment.

**Methods:**

In this randomized trial 60 patients were allocated from September 2011 until March 2013 into one of the two groups: resistance training (group A) or passive physical therapy (group B) with thirty patients in each group during RT. We estimated patient survival using Kaplan-Meier survival method. The Wald-test was used to evaluate the prognostic importance of pathological fracture, primary site, Karnofsky performance status, localization of metastases, number of metastases, and cerebral metastases.

**Results:**

Median follow-up was 10 months (range 2–35). Bone survival showed no significant difference between groups (*p* = .303). Additionally no difference between groups could be detected in overall survival (*p* = .688) and PFS (*p* = .295). Local bone progression was detected in 16.7 % in group B, no irradiated bone in group A showed a local progression over the course (*p* = 0.019). In univariate analysis breast cancer, prostate cancer, and the presence of cerebral metastases had a significant impact on bone survival in group B, while no impact could be demonstrated in group A.

**Conclusions:**

In this group of patients with spinal bone metastases we were able to show that guided resistance training of the paravertebral muscles had no essential impact on survival concomitant to RT. Importantly, no local bone progression in group A was detected, nevertheless no prognostic factor for combined treatment could be evaluated.

**Trial registration:**

Clinical trial identifier NCT 01409720. Registered 8 February 2011.

## Background

Spinal bone metastases represent the most frequent site of skeletal metastasis [1], and two-thirds of all tumor patients are estimated to develop bone metastases in the course of their disease [2]. Bone metastases are a major clinical concern due to severe pain, pathological fractures, spinal cord compression and hypercalcaemia with a significant decrease of the quality of life (QoL) [3, 4]. Radiotherapy (RT) is the most common treatment option of bone metastases in advanced tumor disease [4–6], and is effective in reducing symptoms, increases subjective well-being, and has minimal side effects [7]. The classification into stable and unstable bone metastases and pathological fractures are of great clinical relevance regarding mobility and QoL in patients’ palliative care. Most patients with spinal metastases have a limited life expectancy [8]. The early initiation of therapy, which can generally be viewed as being given as palliative therapy, brings about a significant improvement of the QoL and appears to prolong the survival time [9]. The median overall survival varies from 7 to 32 months, depending on significant predictors e.g., Karnofsky performance score (KPS), primary tumor, and the absence of visceral metastases [8, 10–12]. Previously we showed that within our study guided resistance training of the paravertebral muscles could safety be practiced in palliative patients with stable bone metastases of the vertebral column; leading to an improved pain score and mobility as well as reduced fatigue and thereby an enhanced QoL [13, 14]. Secondary, we were able to show that resistance training concomitant to RT can improve pain relief, and improve bone density in the metastasis as a local response over a 6-months period [15, 16]. However, in our recent work, we analyzed the endpoints feasibility, QoL, local response, and pain of resistance training in patients with spinal bone metastases under RT until 6 months. The aim of this analysis was to compare the bone survival of patients with spinal bone metastases under resistance training versus passive physical therapy concomitant to RT. Secondary endpoints were overall survival, progression free survival, and to quantify prognostic factors to bone survival after combined treatment.

## Methods

This is a randomized, controlled, two-armed intervention trial. A block randomization approach with block size 6 was used to ensure that the two groups were balanced. Inclusion criteria were an age of 18 to 80 years, KPS [17] ≥ 70, written consent to participate, and already initiated bisphosphonate therapy. The patients were subjected to a staging of their vertebral column within the context of the computed tomography (CT) designed to plan the radiation schedule prior to enrolment into the trial. In this examination metastases were classified as “stable” or “unstable”. This was diagnosed independently by a specialist for radiology as well as by a specialist for orthopedic surgery. The specifications for an unstable vertebral body were tumor occupancy more than 60 % of the vertebral body, and pedicle destruction [18]. Only a metastasis classified by both specialists as “stable” was suggested eligible for inclusion. After the baseline measurements, the patients with stable bone metastases were assigned to the respective treatment groups on a 1:1 basis according to the randomization list. Group A (intervention group, resistance training) and in group B (control group, passive physical therapy) each consisted of 30 patients. The primary endpoint was to compare bone survival between the two groups. Secondary endpoints were to quantify overall survival, progression free survival (PFS), and prognostic factors for bone survival. Local bone progression was defined as progressive treated bone metastasis, while systemic progressive bone was defined as additional bone metastases to the treated site. Progressive disease was defined as local progression and/or systemic progressive bone and/or death. PFS was the time between first diagnosis or existence of bone metastases (time equalized to the start of RT) until progressive disease or death. The progression of bone disease was estimated by CT scans 3, 6, 12 and 24 months after RT. The progressive treated bone metastases were classified by MDA criteria [19]. Bone survival was the time from first diagnosis or existence of bone metastases (time equalized to the start of RT) until death, and overall survival was the time from first diagnosis of primary site until death. Bone metastases distant from the irradiated site were not included. Patient-specific data was documented. The study was approved by the Heidelberg Ethics Committee (S-316/2011).

### Radiotherapy

Radiotherapy was performed in the Department of Radiation Oncology at the University Hospital Heidelberg. After virtual simulation was performed for treatment planning, radiotherapy was carried out over a dorsal photon field of 6MV energy range. Primary target volume (PTV) covered the specific vertebral body affected as well as the ones immediately above and below. In group A twenty-four patients (80 %) were treated with 10 × 3 Gy, three patients (10 %) with 14 × 2.5 Gy, and three patients (10 %) with 20 × 2 Gy. In group B the dose fractions for twenty-eight patients (93.3 %) were 10 × 3 Gy, for one patient (3.3 %) 14 × 2.5 Gy, and for one patient (3.3 %) 20 × 2 Gy. The median individual dose in all patients was 3 Gy (range 2–3 Gy), the median total dose 30 Gy (range 20–35 Gy). The individual and total doses were decided separately for each individual patient, depending on histology, patient’s general state of health, current staging and the corresponding prognosis.

### Exercise interventions

The interventions commenced on the same day as radiotherapy and were performed on each day of RT treatment (Monday through Friday) over a 2-week period, independent of the number of fractions. During the 2-week RT period, the patients in the resistance training group (group A) performed the exercises under the guidance of a trained physiotherapist. The patients were then instructed to perform all trainings at home three times per week until the endpoint assessment after 6 months. Self-reported training adherence was registered in a training diary. The resistance training lasted approx. 30 min, the passive physical therapy (group B) approx. 15 min. Since the site of the bone metastases differed from patient to patient, three different exercises were enacted to ensure an even resistance training of the muscles along the entire vertebral column. A detailed description of the exercise interventions was published earlier [16, 20].

### Statistical approach

On account of the explorative character of this study it was not possible to estimate the total number of cases; with a scheduled number of 30 patients per group, it will, however, be possible to detect a standardized mean-value effect of 0.74 with a power of 80 % and an α significance level of 5 %. We calculated descriptive *p*-values of the corresponding statistical tests comparing the treatment groups. Wilcoxon *u* test was used for difference between groups. We estimated patient survival using Kaplan-Meier survival method. Patients were censored on the basis of whether they were alive. The Wald-test was used to evaluate the prognostic importance of pathological fracture (yes/no), primary site (non-small cell lung cancer (NSCLC), breast cancer, prostate cancer, other), KPS (70/>70), localization of metastases (thoracic/lumbar), number of metastases (1/>1), and cerebral metastases. The results were reported as survival times, *p*-values, hazard ratios including 95 % confidence intervals (CI). For all analysis, a *p*-value of 0.05 or less was considered significant. All statistical analyses were done using SAS software Version 9.3 (SAS Institute, Cary, NC, USA).

## Results

From September 2011 through March 2013, consecutively 80 patients with a histologically confirmed cancer of any primary and spinal bone metastases of the thoracic or lumbar segments were considered in the Department of Radiation Oncology at the University Hospital Heidelberg. Fifteen patients were excluded due to unstable metastases, and five patients declined to participate in the study. Sixty patients fulfilled the inclusion criteria and were enrolled into the trial. Groups were balanced at baseline, and except for visceral metastases there were no group differences (Table 1). The median follow-up was 10 months (range 2–35) for both groups. All surviving patients completed all surveys. Mortality did not differ between groups.

**Table 1:** Patient characteristics at baseline

	Intervention group A (*n* = 30)		Control group B (*n* = 30)		*P*-value
	*n*	%	n	%	
Age (years)					
Mean (SD)	61.3 +/- 10.1		64.1 +/- 10.9		0.304
Gender					
Male	14	46.7	19	63.3	0.195
Female	16	53.3	11	36.7	
Karnofsky-index (median, range)	80 (70–100)		80 (70–100)		0.114
Primary site					
Lung cancer	12	40.0	8	26.6	0.320
Breast cancer	5	16.7	6	20.0	0.542
Prostate cancer	5	16.7	9	30.0	0.156
Melanoma	1	3.3	1	3.3	1.000
Renal cancer	1	3.3	2	6.7	0.875
Other	6	20.0	4	13.4	0.325
Localization metastases					0.717
Thoracic	17	56.7	14	46.7	
Lumbar	9	30.0	13	43.3	
Thoracic and lumbar	2	6.7	2	6.7	
Sacrum	2	6.7	1	3.3	
Number metastases					0.257
Mean (range)	1.4 (2–4)		1.7 (1–5)		
Solitary	22	73.3	18	60.0	
Multiple	8	26.7	12	40.0	
Type of metastases					0.781
osteoblast	9	30.0	10	33.3	0.956
osteolytic	21	70.0	20	66.7	0.935
Distant metastases at baseline					
Visceral	12	40.0	5	16.7	0.045
brain	3	10.0	4	13.4	0.688
lung	7	23.3	4	13.4	0.320
tissue	8	26.7	6	20.0	0.542
Pathological fractures	6	20.0	9	30.0	0.371
Hormonotherapy	10	33.3	16	53.3	0.118
Immunotherapy	7	23.3	5	16.7	0.519
Chemotherapy	25	83.3	20	66.7	0.136
Neurological deficit	0	0.0	2	6.7	0.150
Orthopedic corset at baseline	7	23.3	5	16.7	0.519
Radiotherapy dose completed (Gy)					
single dose (median, range)	3 (2–4)		3 (2–4)		1.000
cumulative dose (median, range)	30 (30–40)		30 (30–40)		1.000

**Fig. 1:**
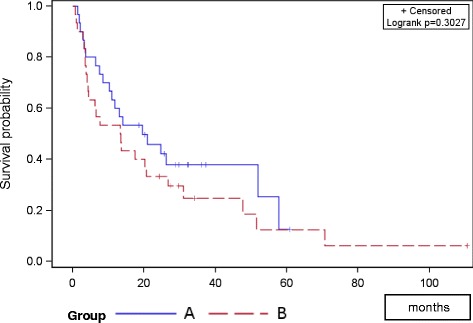
Progression free survival of both groups

**Fig. 2:**
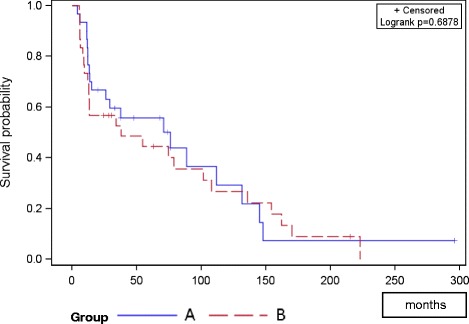
Overall survival of both groups

**Fig. 3:**
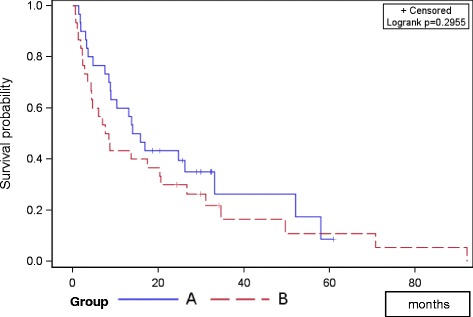
Bone survival of both groups

Bone survival showed no significant difference between groups (*p *= 0.303); bone survival after 12 and 24 months was 58 and 42 % in group A, and 51 and 30 % in group B (Fig. 1). Overall survival after 12 and 24 months was 80 and 63 % in group A, and 70 and 57 % in group B respectively (*p*=0.688; Fig. 2). The PFS did not differ between groups; mean PFS was 24.3 months in group A and 20.5 months in group B (*p* = 0.295; Fig. 3).

Local progression was detected in 16.7 % in group B, no irradiated bone in group A showed a local progression over the course (*p* = 0.019). Progressive disease and systemic bone progression showed no difference between groups (*p* = 0.095 and *p* = 0.108; Table 2).

**Table 2:** Tumor progression of both groups

	Group A		Group B		
	*n*	%	*n*	%	*P*-value
Progressive disease	22	73.3	27	90.0	0.095
Local bone progression	0	0.0	5	16.7	0.019
Systemic bone progression	8	26.7	14	46.7	0.108

In univariate analysis breast cancer (HR 0.103, 95 % CI 0.024–0.442, *p* = 0.002), prostate cancer (HR 0.160, 95 % CI 0.050–0.511, *p*=0.002), and the presence of cerebral metastases (HR 3.211, 95 % CI 1.063–9.695, *p* = 0.038) showed a significant impact on bone survival to group B, while no impact in group A could be demonstrated (Table 3).

**Table 3:** Univariate analysis for prognostic factors of bone survival

	Intervention group (*n* = 30)			Control group (*n* = 30)		
	HR	CI 95 %	*P*-value	HR	CI 95 %	*P*-value
Pathological fracture	1.288	0.371–4.467	0.690	0.833	0.343–2.020	0.686
KPS	0.527	0.216–1.289	0.161	0.872	0.372–2.043	0.752
Localization	0.588	0.209–1.655	0.315	1.166	0.504–2.694	0.720
Number of metastases	0.602	0.200–1.812	0.366	1.052	0.469–2.360	0.902
Breast cancer	0.230	0.028–1.923	0.175	0.103	0.024–0.442	0.002
NSCLC	2.442	0.834–7.145	0.103	0.968	0.346–2.712	0.951
Prostate cancer	0.950	0.237–3.804	0.942	0.160	0.050–0.511	0.002
Cerebral metastases	1.529	0.409–5.716	0.528	3.211	1.063 − 9.695	0.038

## Discussion

The first results of this novel trial showed that guided resistance training of the paravertebral muscles can safely be practiced in palliative patients with stable bone metastases of the vertebral column. Furthermore improved pain and local response, reduced fatigue and enhanced QoL could be detected within 6 months.

In our current analysis, bone survival, overall survival, and PFS showed no significant differences between groups in long-term follow-up. The effect of resistance training showed an improved local response in group A [16], and no local bone progression at the irradiated bone metastases could be detected, while in group B 16.7 % of patients expanded a progression in the vertebral body. Nevertheless these data had no impact on PFS. Overall survival and bone survival showed no differences as well. We interpreted this result as minor impact of resistance training. On the one hand, at baseline visceral metastases were significantly higher in group A, which represents a prognostic factor for survival in the literature, on the other hand a positive tendency for group A could be shown in overall survival and bone survival. Additional small sample size, and different primary tumor types played a major role on the assessment. The most prevalent tumors were those of breast and prostate [21]. These tumor entities had an improved bone survival in group B, but showed no impact in group A. This result explained itself on account of several additional distant metastases which were detected in group A collectively. In a study by van der Linden et al. involving 342 patients with spinal metastases, the most important prognostic factors were performance status, metastatic involvement of other organs and primary site [8]. In the study by Katagiri et al. [22], primary tumor, performance status, number of bone metastases, metastatic involvement of other organs and previous chemotherapy regimens constituted important prognostic factors among 350 patients with bone metastases. In a retrospective analysis of 356 patients, Rades et al. [23] identified that improved survival was significantly associated with female gender, an Eastern Cooperative Oncology Group performance score (ECOG-PS) of 1–2, pre-RT ambulatory status, the absence of other bone metastases, the absence of visceral metastases, an interval from cancer diagnosis to RT of >15 months and slower (>7 days) development of motor deficits. Our trial was not able to demonstrate a survival benefit of resistance training concomitant to RT, and identified only tumor type and cerebral metastases as prognostic factors in our control group. However, the knowledge of prognostic factors and of the prognosis following bone metastasis is of critical importance. A paper by Sugiura et al. [24] considering 118 patients with bone metastases showed a 1-year survival rate of 31.6 % and a 2-year survival rate of 11.3 % for lung cancer. Overall survival rates of patients with renal cell carcinoma were described with 74 % after one year [25]. Correspondingly, the survival rates especially differ in the literature depending on the primary tumor. Based on the different primary types in our trial, these data are not comparable.

Bone metastases are among the most serious problems seen in tumor patients and bone pain, pathological fractures and neurological deficits can be life-threatening events [26]. Palliative RT constitutes one of the most important therapeutic options in these situations. In our recent work, we were able to demonstrate a benefit in QoL, pain response, local response, and reduced fatigue for patients after combined treatment with resistance training concomitant to RT. However, our results showed no differences in survival. In our opinion, the combined treatment with resistance training concomitant to RT is a very effective novel treatment. Future trial designs should stratify to primary tumor and visceral metastases. Further limitations of the study were the relatively small sample size, the variety of primary tumors and patient conditions, and the exclusion of patients presenting with cervical spine metastases. Among the strengths of our novel and original study were the randomized design and long-term follow-up among palliative patients with spinal bone metastases.

## Conclusion

In this group of patients with spinal bone metastases we were able to show that guided resistance training of the paravertebral muscles had no essential impact on bone survival, overall survival, and progression free survival concomitant to RT. Importantly, the absence of local bone progression in group A could be detected, nevertheless no prognostic factor for combined treatment was evaluated.
